# Recent Developments of Fluorescence Sensors Constructed from Pillar[*n*]arene-Based Supramolecular Architectures Containing Metal Coordination Sites

**DOI:** 10.3390/s24051530

**Published:** 2024-02-27

**Authors:** Xu Li, Yan Jin, Nansong Zhu, Jinghua Yin, Long Yi Jin

**Affiliations:** Department of Chemistry, National Demonstration Centre for Experimental Chemistry Education, Yanbian University, Yanji 133002, China; 2023001019@ybu.edu.cn (X.L.); 2022010078@ybu.edu.cn (Y.J.); lyjin@ybu.edu.cn (L.Y.J.)

**Keywords:** fluorescence sensing, environmental pollutants, pillar[*n*]arene, supramolecular architecture, metal coordination complex

## Abstract

The field of fluorescence sensing, leveraging various supramolecular self-assembled architectures constructed from macrocyclic pillar[*n*]arenes, has seen significant advancement in recent decades. This review comprehensively discusses, for the first time, the recent innovations in the synthesis and self-assembly of pillar[*n*]arene-based supramolecular architectures (PSAs) containing metal coordination sites, along with their practical applications and prospects in fluorescence sensing. Integrating hydrophobic and electron-rich cavities of pillar[*n*]arenes into these supramolecular structures endows the entire system with self-assembly behavior and stimulus responsiveness. Employing the host–guest interaction strategy and complementary coordination forces, PSAs exhibiting both intelligent and controllable properties are successfully constructed. This provides a broad horizon for advancing fluorescence sensors capable of detecting environmental pollutants. This review aims to establish a solid foundation for the future development of fluorescence sensing applications utilizing PSAs. Additionally, current challenges and future perspectives in this field are discussed.

## 1. Introduction

Through noncovalent interactions, supramolecular chemistry unites components with different structures and functionalities [1,2,3,4,5,6,7,8], enabling the construction of dynamically reversible smart composites [9,10,11,12]. Increasing attention has been given to intelligent supramolecular structures formed by host–guest interactions, particularly for their stimuli-responsive properties and expansive application potential. As a vital segment of supramolecular chemistry, macrocyclic hosts have found extensive use across various domains. Presently, there are five prominent macrocyclic hosts, including cyclodextrins, calix[*n*]arenes, crown ethers, cucurbit[*n*]urils, and pillar[*n*]arenes. Each host can specifically accommodate different guest molecules by offering suitable cavities. Pillar[*n*]arenes, in particular, stand out due to their electron-rich and rigid cavities, symmetrical frame structure, and exceptional host–guest properties [13,14,15,16,17,18,19,20,21]. The positional modification of pillar[*n*]arenes endows them with distinct characteristics [22,23,24,25,26,27,28]. This review focuses on PSAs that are functionalized with chelating metal ions [29,30].

The coordination metal ions discussed in this review include Ag^+^, Hg^2+^, Cu^2+^, Zn^2+^, Pt^2+^, Fe^3+^, Al^3+^, Eu^3+^, and Tb^3+^. Host–guest complexes with metal coordination sites offer considerable scientific value, characterized by a structure resembling a pillar with many self-assembly driving forces, including cation–π, hydrophobic/hydrophilic, C–H–π, and π–π interactions, among others. We categorize two types of supramolecular architectures with metal ion coordination sites: (i) functional pillar[*n*]arenes with metal coordination sites are initially designed and synthesized. The existing studies on pillar[*n*]arenes derive from the successful synthesis of pillar[5]arenes [31], followed by pillar[6]arenes [32]. Additionally, pillar[*n*]arenes with larger cavities (*n* = 6–15) can be synthesized from pillar[5]arenes by ring expansion, a process driven by kinetic control [33]. Due to the unique advantages of pillar[*n*]arenes, many novel functional pillar[*n*]arenes have emerged one after another [34,35]. The key factors for the design and synthesis of functional pillar[*n*]arene molecules mainly lie in the following two aspects: Firstly, the reaction time, concentration, temperature, feeding order, and other external conditions should be strictly controlled to minimize the production of various polymer byproducts when synthesizing pillar[*n*]arenes. Secondly, considering that the kind, position, and quantity of substituents on both sides of pillar[*n*]arenes have significant effects on their solubility, conformation, and host–guest properties, which directly influences the practical application of pillar[*n*]arenes, it is also challenging to design functional pillar[*n*]arenes. Pillar[*n*]arene derivatives reviewed in this paper mostly concentrate on the single- or dual-functional modifications of the macrocyclic host, including mono, double, and total substitution of the benzene rings. Pillar[*n*]arenes before functionalization often contain halogen atoms, azide groups, hydroxyl groups, or amino groups as the modification sites. Different reaction sites can adopt specific synthesis strategies to achieve high yield, easy separation, green synthesis, and other goals. The pillar[*n*]arenes can be also substituted by molecules (e.g., adenin, quinoline, acylhydrazone, thymine, pyridine, triazole, 1,3,4-oxadiazole, bis-2,2′:6′,2″-terpyridin, carboxyl, naphthalimide, thioacetylhydrazine, et al.) to possess the metal coordination ability. These functional pillar[*n*]arenes can further coordinate with metal ions in the presence or absence of guest molecules, generating supramolecular architectures with specific sensing capacities through multiple noncovalent interaction forces. (ii) Guest molecules with both host–guest recognition abilities and metal coordination sites are firstly designed and synthesized. The host–guest recognition groups are generally composed of quaternary ammonium groups, cyanogen groups, halogen atoms, or nitrogen heterocycles. Additionally, the involved coordination sites mainly consist of phenazine imidazole, thienyl functionalized diketopyrrolopyrrole, or multiple amide groups. After combining these functional groups into a small molecule, these guests can form complexes through host–guest interactions with pillar[*n*]arenes, which further coordinate with metal ions to generate supramolecular architectures with specific sensing capacities.

Environmental pollutants, such as hydrazine hydrate, nitrobenzene, nitroaromatics, cyanide perchlorate, halides, and various metal ions, have been recognized as a critical global issue, posing significant threats to public health and natural ecosystems. Consequently, there is an urgent demand for the development of analytical methods to detect these toxic substances. However, challenges persist across multiple fronts. Analytical methods that offer high sensitivity, exceptional selectivity, adequate removal effectiveness, and a low limit of detection (LOD) are essential, requiring appropriate strategies. Among the methods reported, fluorescence sensing based on supramolecular systems stands out for its accuracy, convenience, and stability, offering superior temporal and spatial resolution, and enabling real-time analysis [36,37,38,39,40]. Aggregation-induced emission (AIE) has emerged as a promising sensing mechanism in recent years. This approach involves materials that exhibit negligible emission in dilute solutions and significantly increased fluorescence in aggregated states [41,42]. The unique host–guest interactions of pillar[*n*]arenes can be harnessed to manipulate AIEgens (aggregation-induced emission generators, indicating smart molecules with AIE properties), leading to supramolecular assembly-induced enhanced emission (SAIEE). This offers the potential to develop new materials with photoluminescent properties. Furthermore, materials based on PSAs containing metal coordination sites have shown promising results in detecting and absorbing heavy metal ion contamination and other pollutants.

Above all, to our knowledge, the recent advancements in fluorescence sensing using PSAs containing metal coordination sites have not been systematically reviewed. This review discusses recent research results, from molecular structure design and luminescence studies to practical application demonstrations in fluorescence sensing. This discussion should inspire further research into self-assembly systems based on PSAs in fluorescence sensing.

## 2. Fluorescence Sensors

Stimulation-responsive fluorescence materials represent a significant category of smart materials. In recent years, these materials have garnered attention due to their tunable luminescence, which can be affected by environmental factors including temperature, light, pressure, guest compounds, etc. In this section, PSAs are systematically categorized and described based on the range of external stimulus conditions they can respond to.

### 2.1. Single-Stimulus Responsive Sensors

**Scheme 1:**
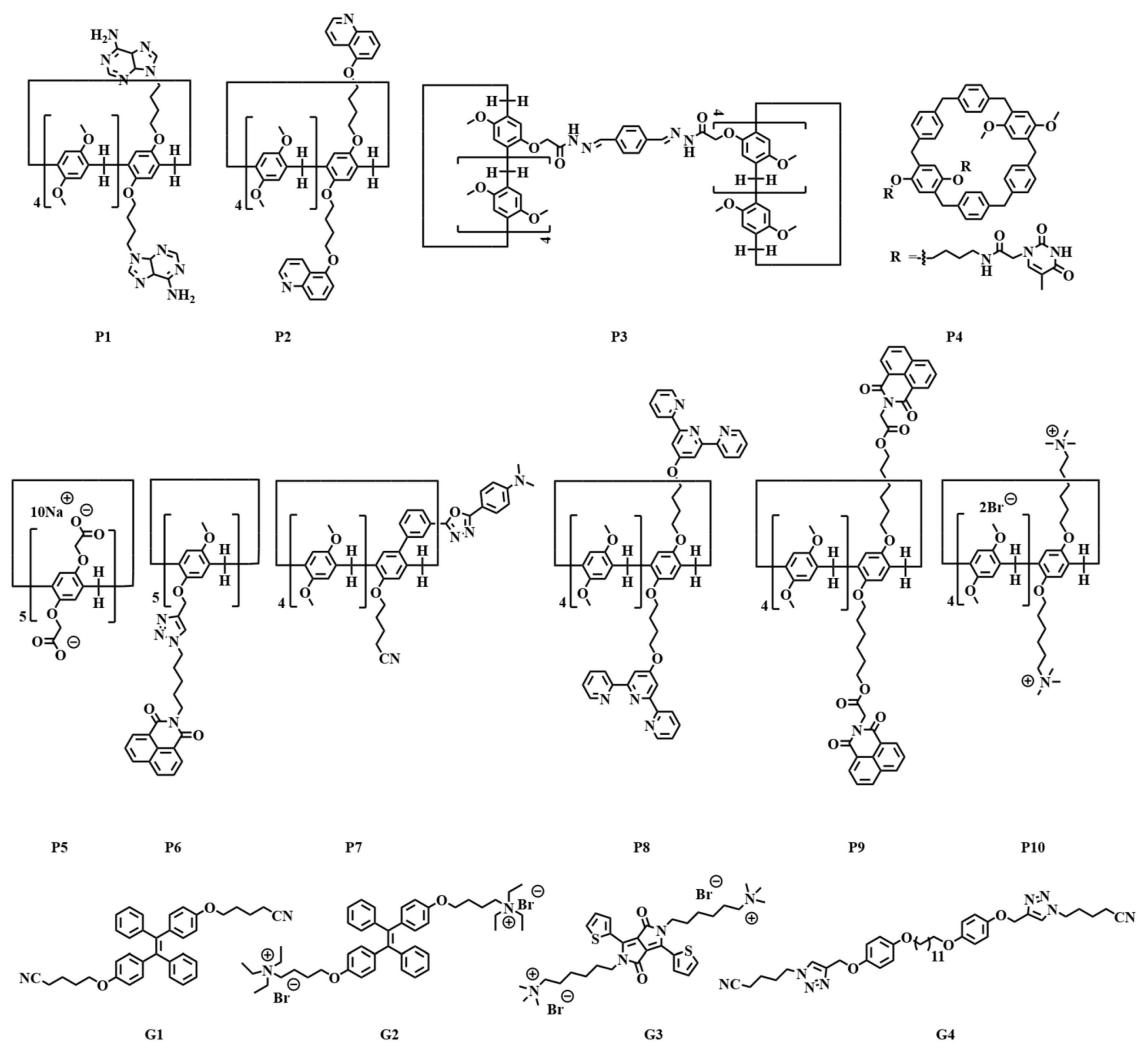
Molecular structures of pillar[*n*]arenes and guests in the section of single-stimulus responsive fluorescence sensors.

Typically, most materials respond to a single stimulus with high sensitivity (Table 1). Scheme 1 lists the molecular structures of the guest molecules and pillar[*n*]arenes that are discussed in this section.

A prime example is the widespread concern over environmental pollution caused by heavy metal ions. Silver(I) (Ag^+^) ions are known for their excellent electrical and thermal conductivity, ductility, and stability. However, excessive silver can disrupt active enzymes by binding to sulfhydryl groups in proteins, leading to their accumulation in the food chain. Therefore, developing economical, simple, environmentally friendly, and efficient materials for detecting and removing Ag^+^ ions is crucial.

**Figure 1:**
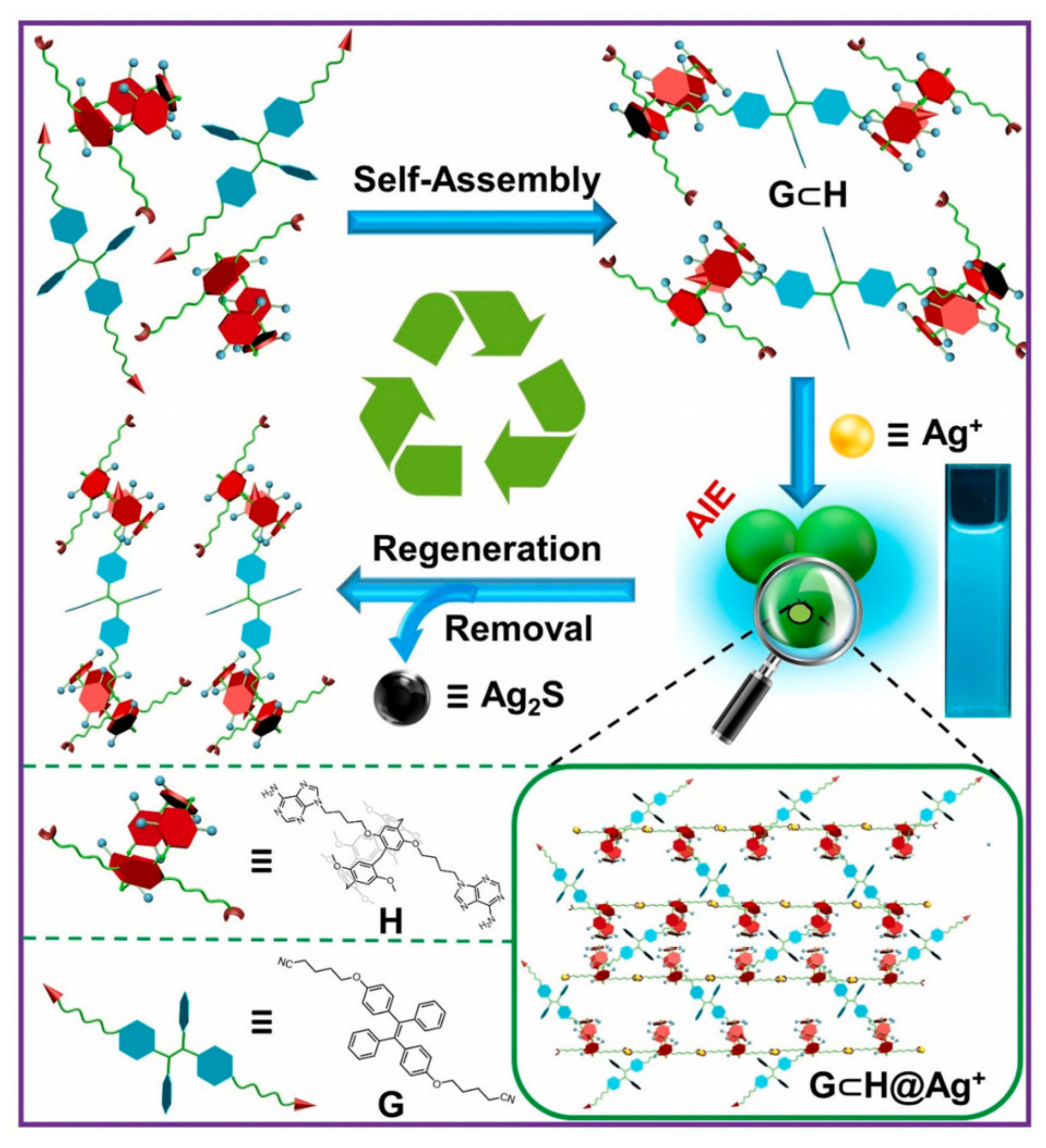
Schematic diagram showing how to detect and remove Ag^+^ ions from water using the supramolecular assembly and the assembly regeneration process [43]. Copyright 2022, Elsevier.

In 2022, Wang W.M. and Yang Y.W. designed a stable supramolecular system utilizing host–guest interactions and coordination effects [43]. They employed two adenine binding sites in pillar[5]arene (**P1**) and tetraphenylvinyl (TPE, **G1**) functionalized with cyano groups, which possess AIE properties (Figure 1). Spherical supramolecular aggregates formed via 1:1 coordination between **P1**’s adenines and Ag^+^ ions. The synergistic effect of coordination between **P1** and Ag^+^, coupled with the host–guest interaction between **P1** and **G1,** resulted in a crosslinked **P1**⊂**G1**@Ag^+^ assembly. This assembly triggered the restriction of intramolecular rotation (RIR) and SAIEE mechanisms. The fluorescence lifetime and fluorescence quantum yield of **P1**⊂**G1**@Ag^+^ were 4.60 ns and 58.38%, respectively, with the rate constant of nonradiative decay being half that of **P1**⊂**G1**. Under 365 nm UV lamp irradiation, the assembly displayed bright blue-green fluorescence upon Ag^+^ ion addition, permitting effective Ag^+^ ion adsorption and sensitive detection. Moreover, the supramolecular assembly could be easily processed without a decline in activity, offering a useful tool for practical applications.

The need for effective devices for sensitive toxic gas detection is also paramount. For instance, hydrazine hydrate (DH) is extensively used in synthesizing pesticides and medicines, fuels for satellites and rockets, and as a preservation agent in nuclear and power facilities. However, DH is very harmful, causing severe damage to the skin and central organs upon human absorption. Consequently, developing flexible, fast, and sensitive materials and methods for DH detection is critically important.

**Figure 2:**
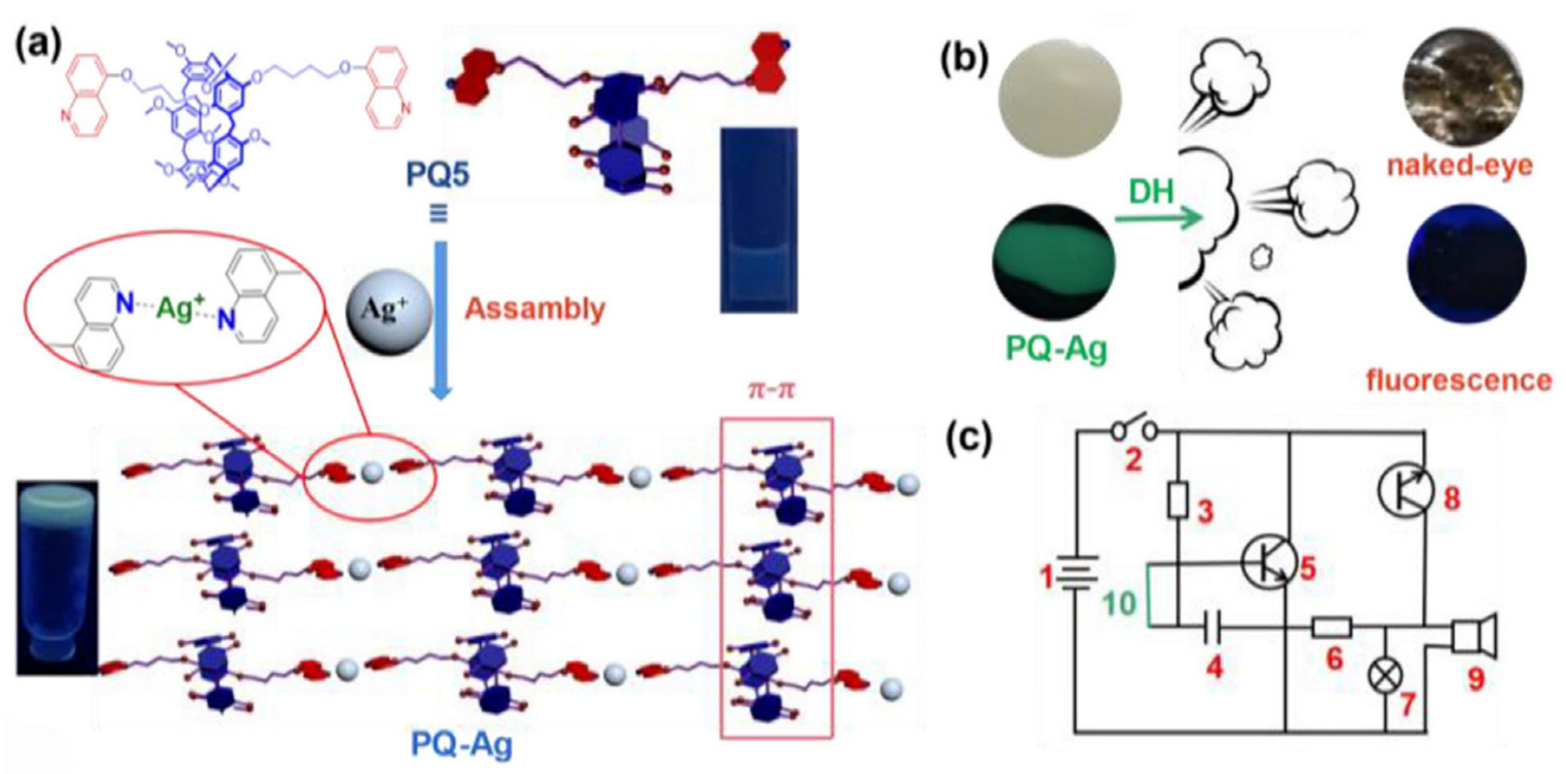
(**a**) A cartoon depicting the metallo-supramolecular polymer gel’s assembly method, (**b**) the PQ-Ag’s multichannel DH detection, and (**c**) the DH alarm circuit schematic diagram (1: electric source; 2: switch; 3: resistance; 4: electric capacity; 5: NPN triode; 6: resistance; 7: bulb; 8: PNP triode; 9: buzzer; 10: gel probe) [44]. Copyright 2023, Elsevier.

A year later, Lin Q.’s group developed a metallic gel by coordinating the nitrogen atom of 5-hydroxyquinoline functionalized pillar[5]arene (**P2**) with Ag^+^ ions [44]. **P2** formed a one-dimensional coordination polymer through interaction with Ag^+^ ions via the N atoms on the quinoline groups (Figure 2). Adjacent coordination polymers were interconnected through π–π interactions between the **P2** groups, facilitating the metallogel’s formation. The mean squared displacement of the gel **P2** was lower than that of the **P2**-Ag gel, indicating that the inclusion of Ag^+^ improved the flexibility of the **P2**-Ag gel. The supramolecular assembly was further analyzed from a microscopic morphology perspective. **P2** alone exhibited a lamellar structure. After adding Ag^+^ ions, the resultant metallogel **P2**-Ag transformed into a folded membrane structure, which is attributable to the coordination bond between Ag^+^ ions and **P2**. When exposed to DH vapor, the folded membrane structure of **P2**-Ag altered into a microspherical structure, suggesting the disruption of Ag^+^ ion coordination and the formation of a microspherical structure by **P2** based on the hydrophobic effect. This gel enabled multichannel sensitive detection of DH through visual, fluorescence, and electrochemical means. DH disrupted the coordination by reducing Ag^+^ ions under DMSO/H_2_O conditions (f_w_ = 20%), leading to the collapse and fluorescence quenching of the metallogel. The lowest critical gelation concentration (CGC) was 8% (*w*/*v*, 10 mg/mL = 1%). The gel–sol transition temperature was 62–63 °C. Multichannel detection of DH could be conveniently and efficiently realized in both water and air through sound and light alarms. The LOD reached 0.10 mg/m^3^ in air and 2.68 × 10^−8^ mol/L in water, below the US Environmental Protection Agency’s standard for drinking water.

Mercury (Hg^2+^), among the most hazardous heavy metals, poses significant health and environmental risks. Exposure to Hg^2+^ ions, even in minute concentrations, presents a potential hazard to humans. For instance, in 2018, the group led by Lin Q. designed and synthesized another sensor based on pillararene AIEgens, which utilized bi-pillar[5]arene-based assemblies incorporating advanced AIEgens (**P3**) [45]. The assembly was driven by intermolecular hydrogen bonding (such as –N–H⋯C=O– and –C–H⋯N=C–), π–π stacking interactions, and hydrophobic effects. The Tyndall effect was observed in a 30% aqueous solution, with the critical aggregation fraction of water being 24% for **P3**, which exhibited a fluorescence quantum yield of 21%. **P3** formed a sharp rod-shaped structure in DMSO/H_2_O (f_w_ = 50%). The aggregated **P3** was disassembled at low concentrations or high temperatures. A 1:2 complex was formed by binding **P3** with Hg^2+^ ions (binding constant: 2.50 × 10^3^ L^2^/mol^2^). The coordination between the Hg^2+^ ions and the **P3** acylhydrazone group served as the basis for the sensing mechanism. To explore practical applications, a glass sheet was submerged in a high concentration of **P3** to generate a film. This film enabled the convenient detection of Hg^2+^ ions in water, effectively separating and sensitively detecting them with an LOD of 4.30 × 10^−8^ mol/L. This innovative bi-pillar[5]arene AIEgen could pave the way for new designs and developments in pillar[*n*]arene AIEgens.

**Figure 3:**
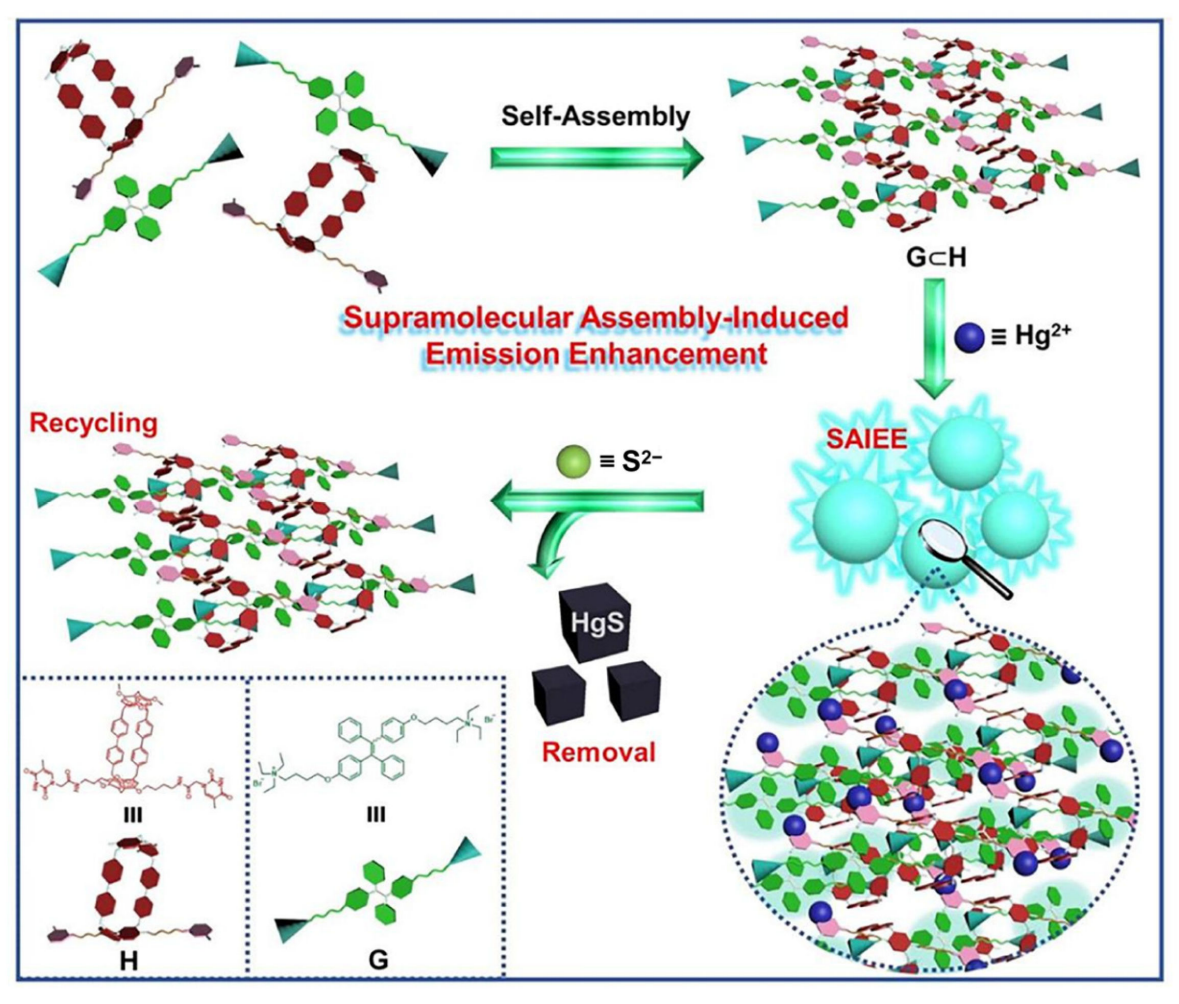
Schematic illustration showing how to detect and remove Hg^2+^ ions from water using the regeneration–recycling process and the “switch-on” fluorescence of supramolecular polymers [46]. Copyright 2019, American Chemical Society.

The pillar[*n*]arenes discussed in this paper include two types: pillar[5]arene and pillar[6]arene [46]. The latter was developed by Yang Y.W.’s group. They utilized the strong interactions between thymine (T) and Hg^2+^ ions. Building on this, Dai D.H. and Yang Y.W. constructed a crosslinked supramolecular polymer through host–guest interactions, utilizing a TPE-bridged bis(quaternary ammonium) guest (**G2**) with AIE characteristics and a newly constructed [2]biphenyl-extended pillar[6]arene with two thymine sites as arms (**P4**) (Figure 3). The thymine groups’ close T-Hg^2+^-T coupling with the Hg^2+^ ions led to the formation of spherical assemblies with an average diameter of 164 nm. Fluorescent emission occurred immediately upon the addition of Hg^2+^ ions. The introduction of Hg^2+^ ions into the supramolecular system initiated supramolecular SAIEE. With its integrated mode of operation, great selectivity, and a quick adsorption rate (removal efficiency: 90%), this supramolecular polymer effectively accomplished the real-time detection and removal of Hg^2+^ ions from water. The assembly of **P4** and **G2** demonstrated excellent recyclability, maintaining effectiveness over more than five cycles of the removal process.

**Figure 4:**
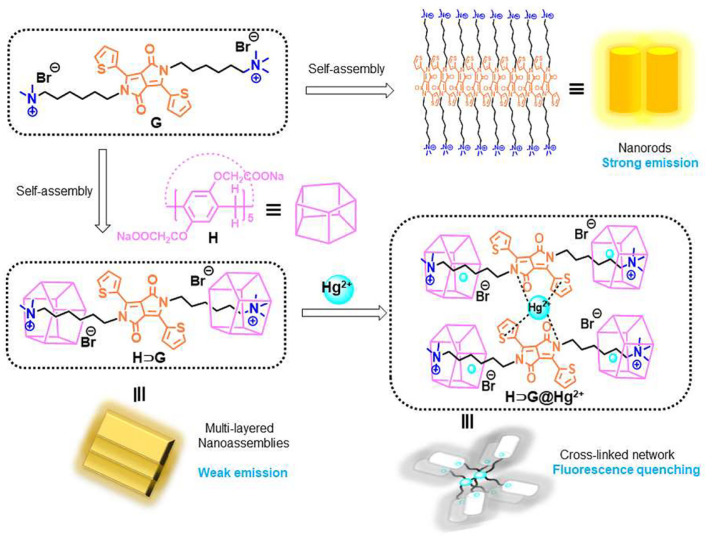
Schematic representations of the self-assembly of carboxylatopillar[5]arene sodium salts in the presence and absence of Hg^2+^ ions in water, as well as chemical structures and cartoon representations of the diketopyrrolopyrrole-bridged bis(quaternary ammonium) guest [47].

Beyond the contributions of the previously mentioned research groups, a self-assembly consisting of carboxylatepillar[5]arene sodium salts (**P5**) and a diketopyrrole-bridged bis-quaternary ammonium guest (**G3**) was reported by Jiang X.M. and Cao D.R. [47]. Adding **P5** to a **G3** aqueous solution resulted in the morphological transformation of multilayer nanostructures (Figure 4). This host–guest complex exhibited multiple forces, including electrostatic, hydrophobic interactions, and π–π stacking interactions in aqueous solutions. The developed supramolecular system effectively detected and removed Hg^2+^ in real environmental water samples. The Hg^2+^ ions demonstrated synergistic interactions, including coordination with **G3** and **P5** and the Hg^2+^-cavity, forming a crosslinked network of **P5**⊂**G3**@Hg^2+^. The method exhibited good selectivity with a low LOD of 7.17 × 10^−7^ mol/L. Furthermore, the quenched fluorescence could be recovered post-treatment with Na_2_S, exhibiting a reversible process.

Copper ions (Cu^2+^) are also critical in various industries, and can result in soil contamination, bioaccumulation, and decreased agricultural production. Therefore, developing a selective and sensitive Cu^2+^ ion fluorescent chemical sensor, preferably a proportional chemical sensor, remains crucial in ion sensing research.

Pillar[5]arene was modified by Chang R. and Chang K.C. with five neighboring naphthalimide groups to form a new ligand, **P6**, for metal ion coordination, effectively serving as a ratiometric fluorescence sensor for Cu^2+^ ions in a CH_2_Cl_2_/CH_3_CN = 1/1 system [48]. This sensitivity was also observed in 10% aqueous methanol solutions. The introduction of naphthalimide groups enhanced intramolecular π–π stacking. **P6** exhibited dual emission, comprising both the monomer and excimer emissions of the naphthalimide moieties. A synergistic interaction occurred between Cu^2+^ ions and the triazole groups on **P6**. **P6** rapidly bound with Cu^2+^, maintaining stable fluorescence intensity. Upon complexing with Cu^2+^ ions, the excimer emission of ligand **P6** was weakened, while the monomer emission intensified. The binding complexation ratio of **P6** with Cu^2+^ ions was 1:1, with a binding constant of (3.39 ± 0.40) × 10^5^ L^2^/mol^2^ and an LOD of 1.85 × 10^−6^ mol/L. Particles varying in diameters from (192 ± 65) to (206 ± 67) nm were produced. The relative fluorescence quantum yields of **P1** and **P1**-Cu^2+^ were 0.13 and 0.11, respectively. The pillar[5]arene framework may be further functionalized in future studies to improve its selectivity for particular metal ions, or to modify it for use with different sensing platforms.

Liu S.Y. and Han J. developed another sensor for Cu^2+^ ions [49]. They designed a pillar[5]arene framework by functionalizing it with a cyanobutoxy moiety (P7) and a 1,3,4-oxadiazole subunit. This structure facilitated host–guest interactions between the electron-rich pillar[5]arene cavities and appropriately-sized neutral cyanobutoxy moieties, resulting in brush supramolecular polymers. Notably, the larger electron-deficient 1,3,4-oxadiazole groups remained outside the pillar[*n*]arene (**P7**) cavity after the creation of the host–guest inclusions, acting as a “brush” and enhancing the ability of the self-assembled supramolecular materials to interact with metal ions, such as Cu^2+^ ions. The critical aggregation concentration of **P7** was 6.00 × 10^−2^ mol/L. These supramolecular brush-polymer architectures displayed distinct structural changes in response to fluorescence quenching after adding Cu^2+^ ions, suggesting a potential transformation into a crosslinked supramolecular network. The Irving–Williams order of stability might provide an explanation for the particular recognition of Cu^2+^. Therefore, this supramolecular brush polymer holds potential for application in metal cationic fluorescent chemical sensors.

In 2021, Chong H. et al. prepared a “three-component” supramolecular assembly by combining terpyridine attached pillar[5]arene (**P8**), cyano- and triazole-bearing alkyl chain (**G4**), and Zn^2+^ ions in a CHCl_3_ and CH_3_CN solvent system [50]. Terpyridine’s strong affinity for a variety of transition metal ions makes it an adaptable building block. The resulting composition exhibited a closely crosslinked porous morphology with nanoscale pore size. The **P8** segment was incorporated into the cyano and triazole segment upon encapsulation. An organogel formed at a concentration of 1.00 mol/L. The polymerization was driven by host–guest interaction and metal-chelate cooperative forces. The **P8** and Zn^2+^ assembly had a flake-like morphology. Nitrobenzene (picric acid, o-nitrobenzene, and phenol) was used as a sample. Of the three analytes, the assembly exhibited the highest sensitivity to picric acid, with an LOD of 1.66 × 10^−4^ mol/L. The quenching mechanism was believed to involve mixed processes of photo-induced electron transfer (PET) and fluorescence resonance energy transfer (FRET). This study represents a practical illustration of creating functional “multi-component” supramolecular systems with capabilities for explosive detection.

The role of Fe^3+^ ions as an essential metal element in human physiology is well-recognized. However, both deficiency and excess of Fe^3+^ ions beyond permissible limits can lead to severe health issues, such as anemia, tumorigenesis, organ dysfunction, and neurodegenerative diseases such as Alzheimer’s and Parkinson’s. Consequently, sensitive detection of Fe^3+^ ions is a critical concern in environmental and health monitoring. In 2018, Zhang Y.M. and Qi L. discovered that the pillar[5]arene structure (**P9**) could self-assemble into high-molecular-weight supramolecular π-gels with AIE properties [51]. Notably, the simplicity of the supramolecular system components enhanced its practical application value. Through π–π stacking interactions, **P9** self-assembled into one-dimensional linear supramolecular π–gel chains. These π–gel chains interacted with the pillar[5]arene and naphthalimide to produce a two-dimensional supramolecular network. Subsequently, hydrophobic interactions compressed the two-dimensional network into microspheres. The CGC was 5% (*w*/*v*, 10 mg/mL = 1%). The gel–sol transition temperature was 43–45 °C. The LOD was 6.06 × 10^−8^ mol/L for Fe^3+^ ions. The gel demonstrated excellent recyclability and a 99.80% removal rate for Fe^3+^ ions in aqueous solutions. Additionally, the Fe^3+^ ion-coordinated supramolecular gel selectively sensed L-Cys with an LOD of 1.00 × 10^−8^ mol/L. This gel could be utilized in logic gates, offering significant advantages over previous logic devices in terms of reversibility, sensitivity, and prospective uses for ongoing transition metal and amino acid detection.

Because of their vital functions in chemical, biological, and environmental applications, the development of ultrasensitive materials and reactions has received a lot of attention recently. In light of this, the above-mentioned group continued to enhance the sensitivity and stability of sensor materials based on their prior findings. They achieved an LOD for Fe^3+^ ions detection reduced from 10^−8^ to 10^−9^ magnitude [52]. They specifically created a new supramolecular organic framework (SOF) that is based on two pillar[5]arene molecules: **P9**, which is functionalized with bis-naphthalimide, and **P10**, which is functionalized with bis-ammonium. Through the competition of cation–π and π–π interactions, this SOF developed a persistent supramolecular gel in cyclohexanol, enabling an ultrasensitive response to Fe^3+^ ions. Upon adding 0.50 equivalent of Fe^3+^ ions, the yellow fluorescence of the gel was quenched with an LOD of 7.54 × 10^−9^ mol/L. The Fe^3+^ ion-coordinated gel could sense H_2_PO_4_^−^ ions with a fluorescence “turn-on” response and an LOD of 4.21 × 10^−9^ mol/L. Response films were prepared using these soft gels, which could also function as sensitive logic gates, fluorescent display materials, and ultra-sensitive ion sensors.

### 2.2. Dual-Stimuli Responsive Sensors

**Scheme 2:**
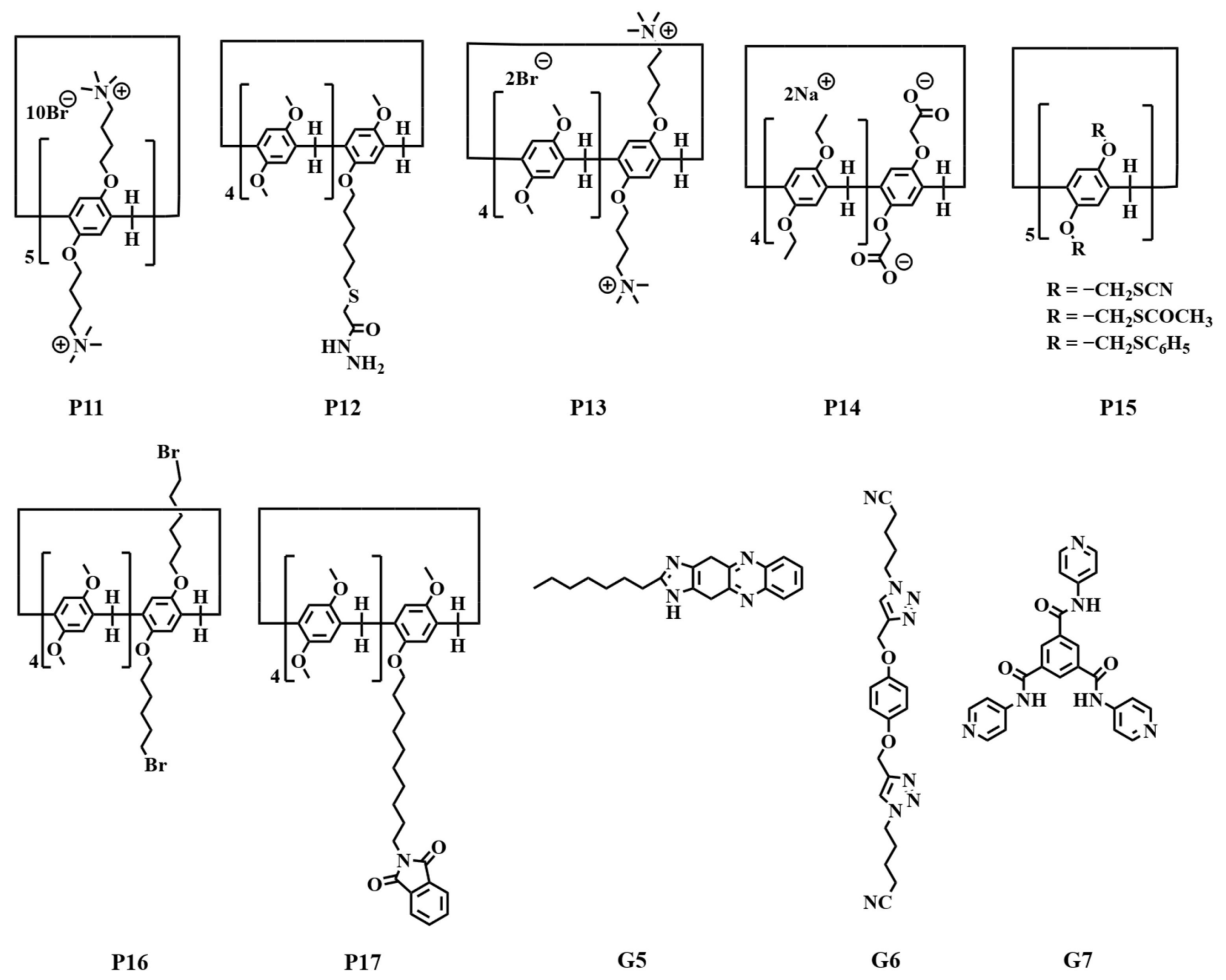
Molecular structures of pillar[*n*]arenes and guests in the section of dual-stimuli responsive fluorescence sensors.

The development of dual-response materials (Table 2) for complex systems is of great significance and challenge. The molecular structures of pillar[*n*]arenes and guest molecules associated with these smart materials are listed in Scheme 2.

In 2018, the group of Zhang Y.M. and Wei T.B. constructed an advanced bifunctional supramolecular pseudorotaxane chemosensor using host pillar[5]arene **P11** and guest **G5** [53]. Adding Ag^+^ and Hg^2+^ ions to this system resulted in fluorescence quenching and significant changes in fluorescence color, respectively. This method demonstrated high sensitivity and selectivity, with LODs of 1.20 × 10^−8^ and 5.00 × 10^−7^ mol/L for Ag^+^ and Hg^2+^ ions, respectively.

**Figure 5:**
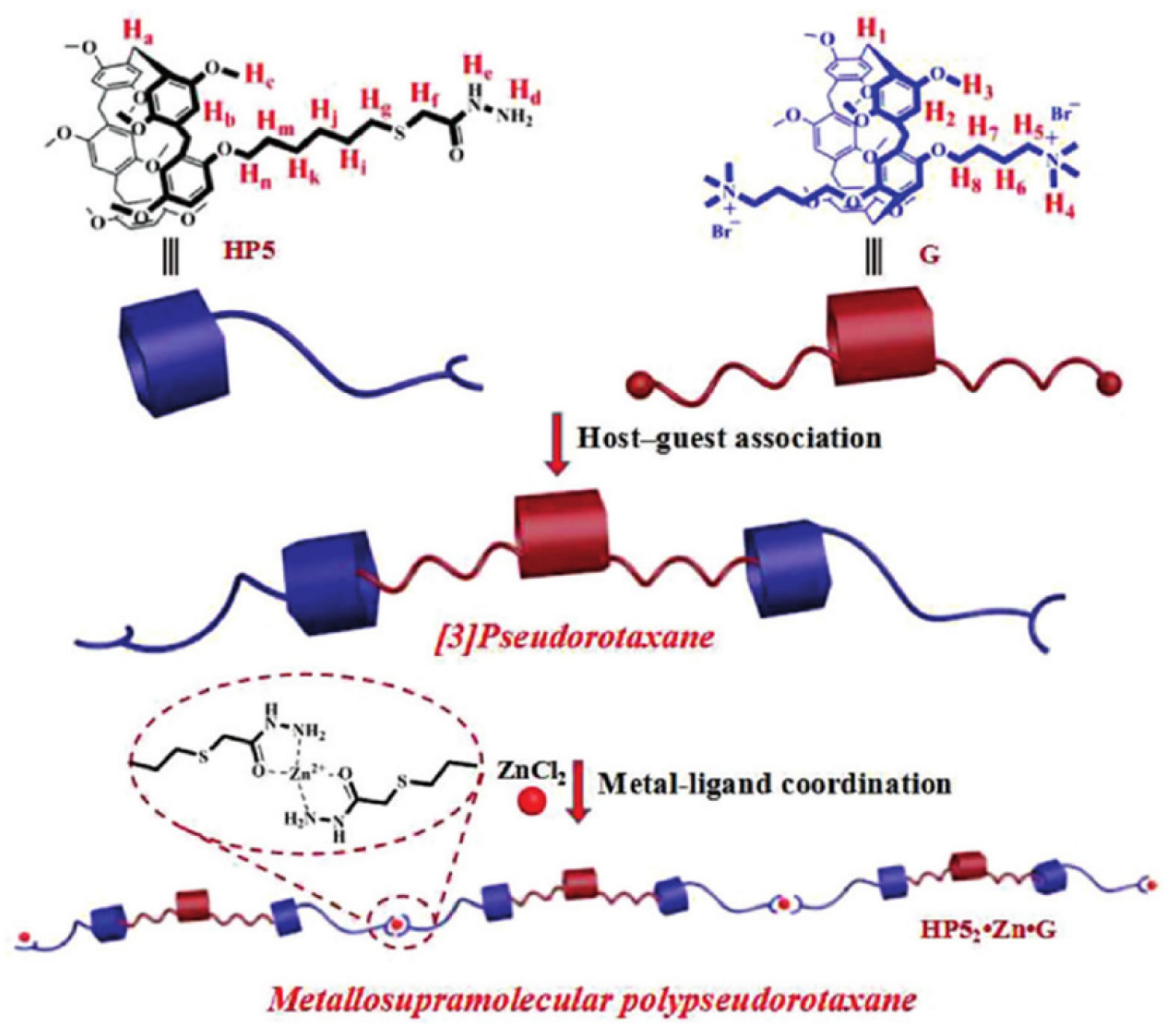
HP5 and G’s chemical structures, proton designations, and a cartoon depicting the creation of the linear metallo-supramolecular polypseudorotaxane framework [54]. Copyright 2018, The Royal Society of Chemistry.

The group led by Wei T.B. [54] developed a novel metallosupramolecular polypseudorotaxane by integrating thioacetylhydrazine functionalized pillar[5]arene (**P12**) and bis-butyltrimethyl ammonium functionalized pillar[5]arene (**P13**) with Zn^2+^ ions (Figure 5). The hydrazyl groups served as coordination and hydrogen bond sites for self-assembly with Zn^2+^ ions. This gel acted as an ultrasensitive fluorescence sensor for detecting Fe^3+^ and Cu^2+^ ions, with LODs of 8.93 × 10^−10^ and 4.57 × 10^−8^ mol/L, respectively. Thin films based on this gel could be used as convenient test kits. Furthermore, this study presented a simple and effective strategy for achieving multiple stimulus responses by introducing multiple noncovalent interactions in metal supramolecular gels.

**Figure 6:**
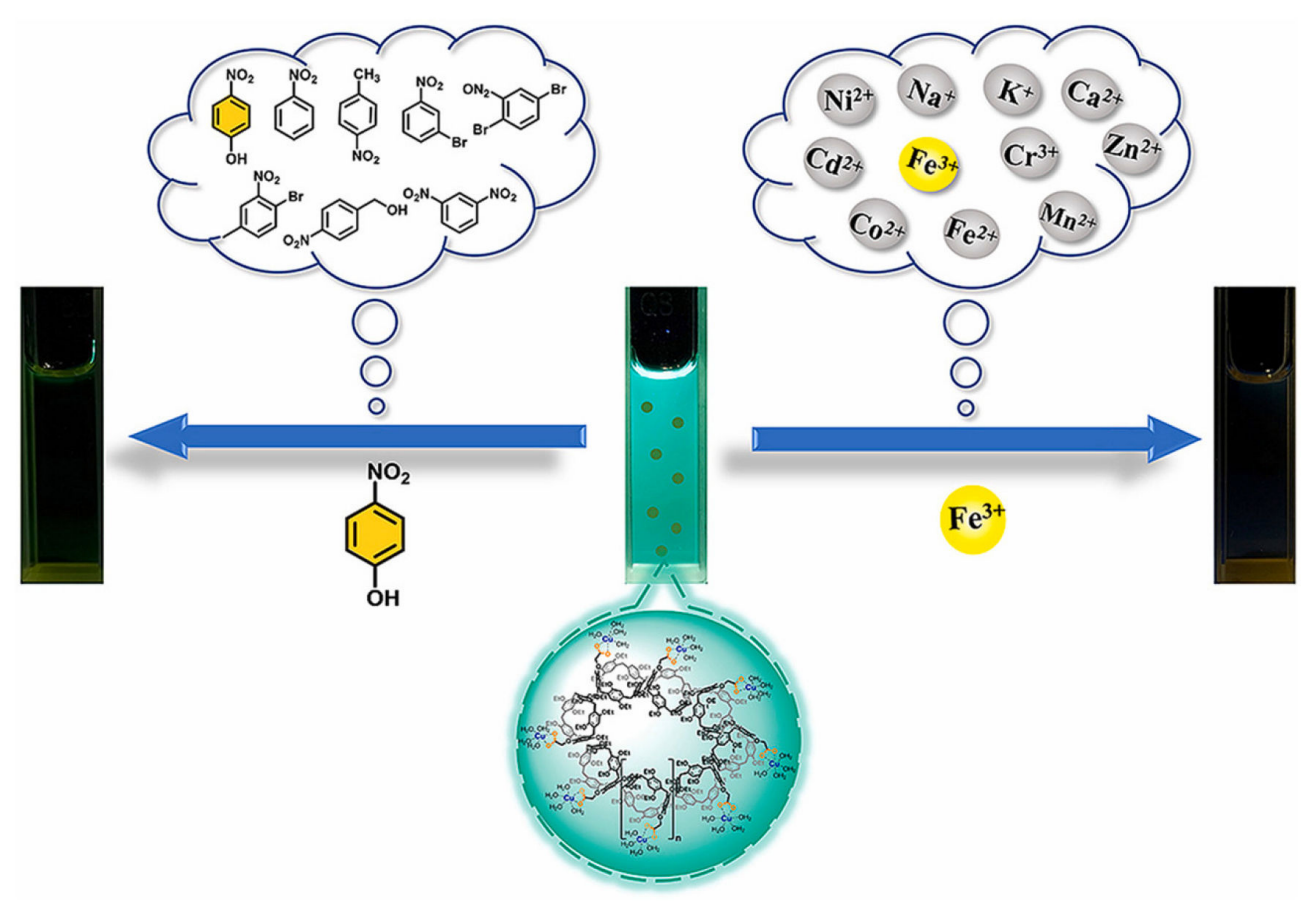
Diagrammatic representation of the pillar[5]arene-based Cu(II) coordination supramolecular aggregate’s structure, as well as p-nitrophenol (PNP) and Fe^3+^ ion detection capabilities [55]. Copyright 2022, Elsevier.

In the context of escalating environmental pollution and its threats to human health and safety, nitroaromatic compounds (NACs) have been identified as major pollutants. NACs are widely utilized in the chemical, dye, explosive, pesticide, and medicinal sectors. P-nitrophenol, for example, can result in major health problems such as methemoglobinemia, fever, liver, and kidney damage. In 2023, Yu D.Y., Deng W.T., and Wei X.Q. synthesized a blue-green fluorescent supramolecular aggregate capable of self-assembling from pillar[5]arene-based Cu^2+^ ions coordination complexes [55]. Two carboxyl O atoms from one **P14** molecule and four O atoms from four water molecules made up the octahedral coordination of the Cu^2+^ ions (Figure 6). These coordination complexes were self-assembled into spherical aggregates through multiple noncovalent forces, including hydrophobic effects, van der Waals forces, and CH–π interactions. The aggregate exhibited high sensitivity and selectivity to nitroaromatic compounds and metal ions. The fluorescence emission of the aggregate was specifically and significantly quenched by p-nitrophenol and Fe^3+^ ions, with low LOD values of 3.90 × 10^−7^ and 4.90 × 10^−6^ mol/L, respectively. Additionally, due to their exceptional performances, these coordinated supramolecular aggregates based on pillar[*n*]arenes hold promise for various applications in adsorption, sensing, cargo transport, and catalysis.

**Figure 7:**
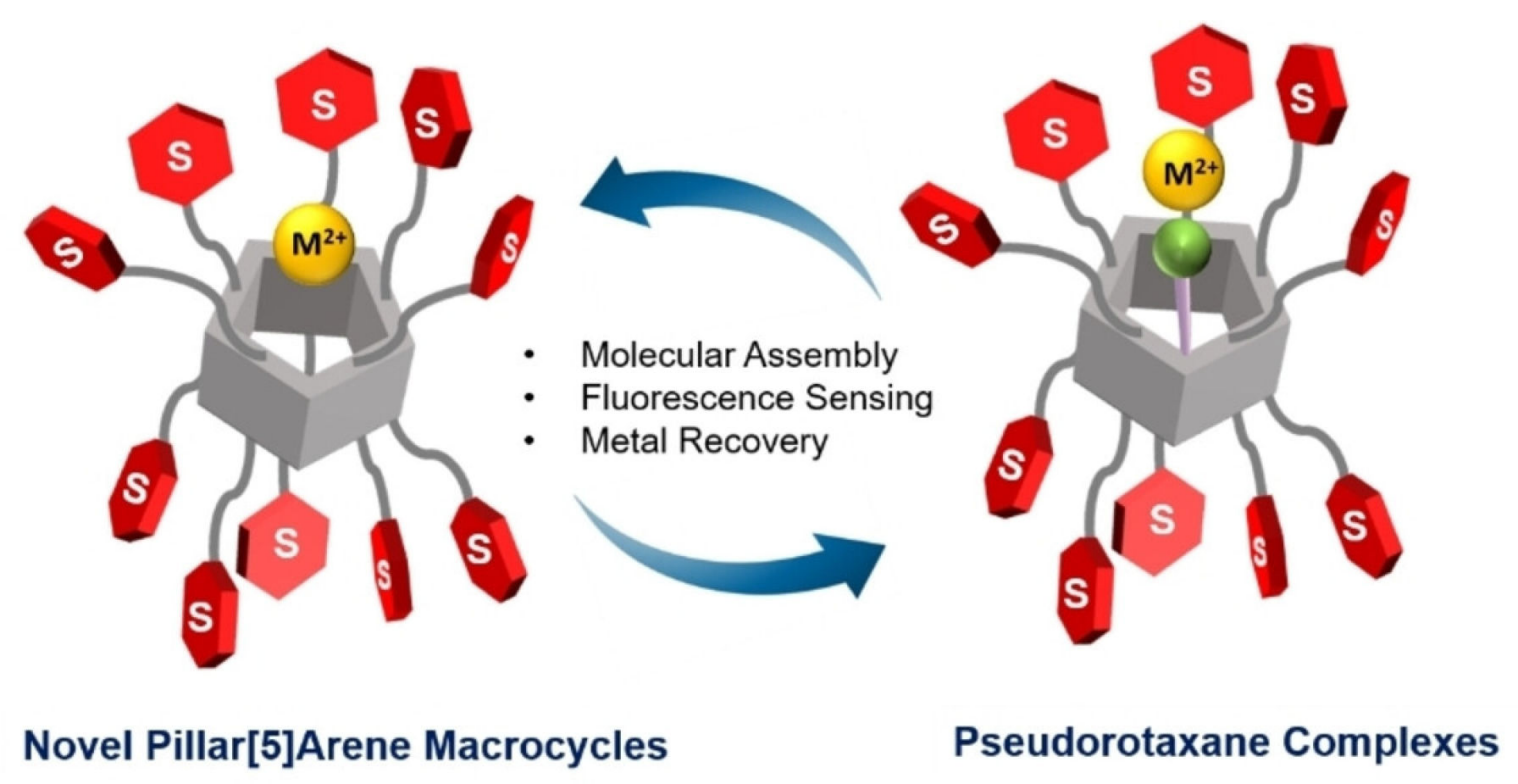
Cartoon illustrating functionalized pillar[5]arenes with sulfur substituents and associated pseudorotaxane complexes [56].

Sulfur substituent-decorated pillar[5]arene (**P15**) showed control over binding affinities toward encapsulations of organic guests inside its cavity [56]. The exterior cavity of **P15** tended to form metal-ion coordination bonds (Figure 7). Adding a dinitrile guest (**G6**) containing a bis-triazole benzene spacer led to the creation of pseudorotaxane host–guest complexes. The fluorescence emission signals were significantly reduced in the presence of Hg^2+^ and Cu^2+^ ions. These macrocycles facilitated the transfer of Cu^2+^ ions from the aqueous to the organic phase, enhancing efficiency compared to extraction processes without pillar[5]arene.

Zhang Y.M. and Lin Q. created a unique SOF gel using pillar[5]arene host (P2) and a bilateral bromohexyl functionalized pillar[5]arene guest (**P16**) [57]. The SOF gel was constructed through CH–π, CH–O, and “exo–wall π–π” interactions between **P2** and **P16**. The introduction of Fe^3+^ ions into the gel resulted in quenched fluorescence. The gel exhibited an LOD of 1.02 × 10^−10^ mol/L for Fe^3+^ ions. The coordinated metallogel of Fe^3+^ ions exhibited a fluorescent “turn-on” response due to the competitive coordination of F^−^ anions, having an LOD of 9.79 × 10^−9^ mol/L. The xerogel of this SOF gel demonstrated an adsorption rate of 99.90% for removing Fe^3+^ ions. This SOF gel and the Fe^3+^ coordinated metallogel served as an ultrasensitive and reversible fluorescence sensor and test kit for Fe^3+^ ions and F^−^ anions.

Toxic contaminant ion detection is always a significant problem. Many anions are harmful to human health. Perchlorates (ClO_4_^−^ anions) disrupt the production and secretion of thyroxine and, therefore, affect the normal metabolism and metabolism of the human body. Cyanide (CN^−^ anion) is a highly toxic compound, and is common in industry and found in nature. Therefore, it is crucial to monitor and eliminate these harmful anions. Zhang Q. and Lin Q. have been actively working to address these challenges [58]. The powder of **P17** displayed a rod-like structure, while **G7** formed a blocky structure. **P17** and **G7** assembled via π–π interactions to create a xerogel structure with a crosslinked network. This xerogel exhibited strong yellow AIE properties. It demonstrated the ability to coordinate the rare earth metal ions Eu^3+^ and Tb^3+^, enabling it to detect ClO_4_^−^ and CN^−^ anions through a fluorescence “turn-on” mode. Additionally, the xerogel demonstrated efficient removal capabilities for these two anions.

### 2.3. Multi-Stimuli Responsive Sensors

**Scheme 3:**
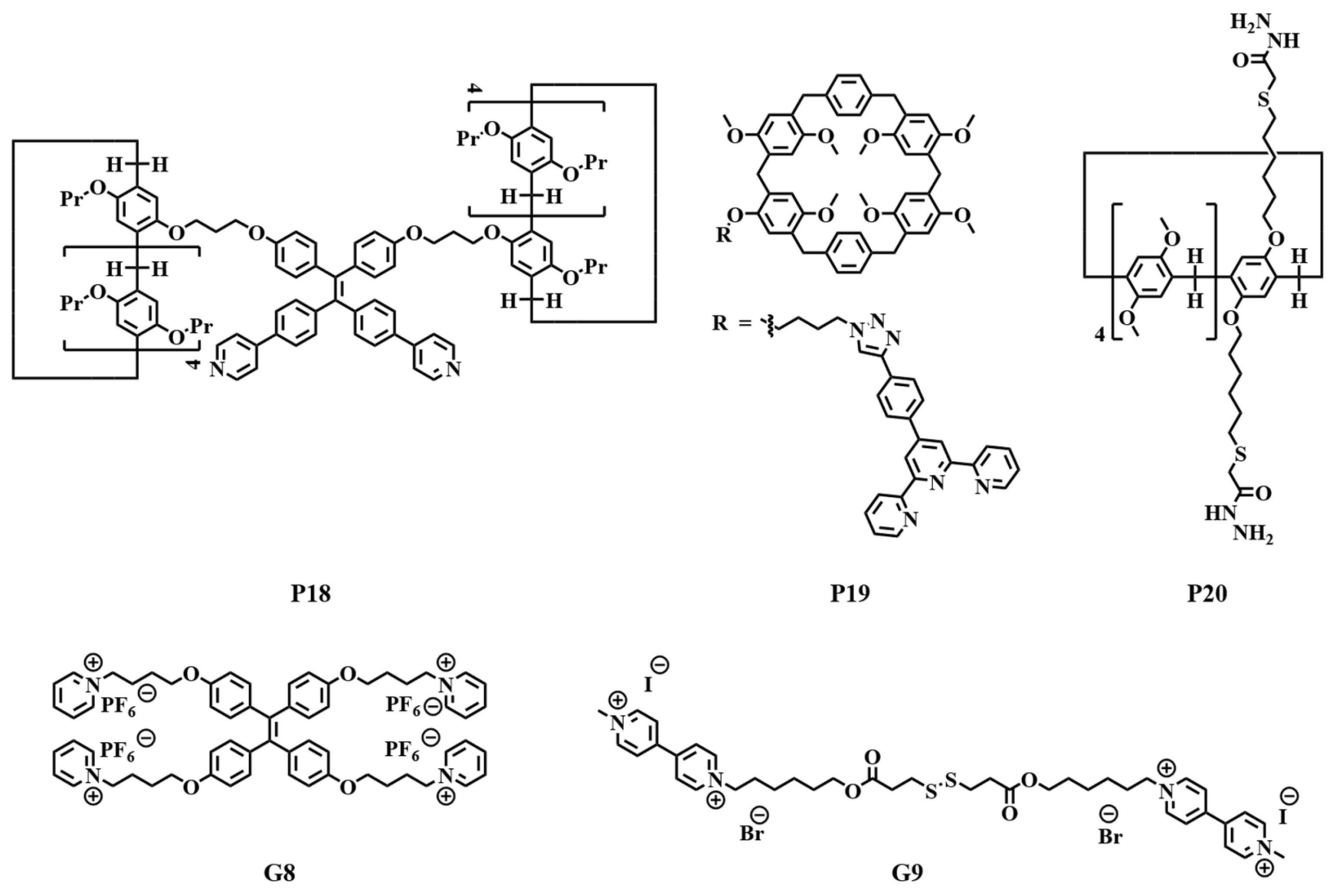
Molecular structures of pillar[*n*]arenes and guests in the section of multi-stimuli-responsive fluorescence sensors.

To accomplish multi-stimuli-responsive detection in complex environments, it is crucial to continuously explore various units to construct novel multi-stimuli-responsive fluorescence materials. The sensing capacities of these materials are presented in Table 3, and the molecular structures of pillar[*n*]arenes and guest molecules newly mentioned in this section are listed in Scheme 3.

**Figure 8:**
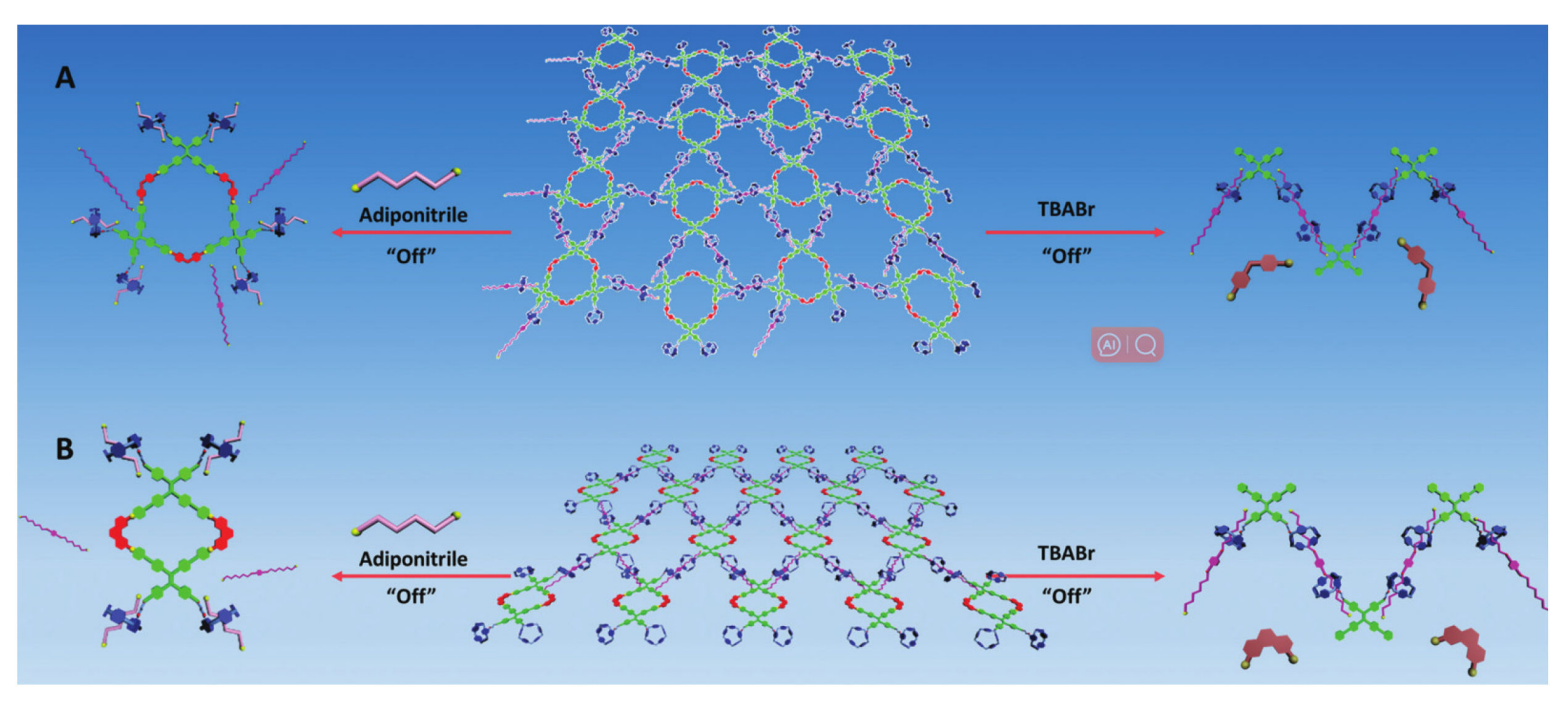
Diagrammatic depiction of the disassembly of supramolecular polymer gels caused by the bromide anion and competitive guest stimuli [59]. (**A**) The gel assembled by the hexagonal metallacycle with six pillar[5]arene units. (**B**) The gel assembled by the rhomboidal metallacycle with four pillar[5]arene units. Copyright 2018, The Royal Society of Chemistry.

Yang H.B.’s group synthesized a dipyridyl donor (**P18**) containing a TPE scaffold and two pillar[5]arene units [59]. **P18** exhibited broad absorption bands at a wavelength of 336 nm with molar absorption coefficients of 2.10 × 10^4^ L·mol^−1^·cm^−1^. Utilizing **P18**, they constructed a rhomboidal metallacycle with four pillar[5]arene units and a hexagonal metallacycle with six pillar[5]arene units (Figure 8). These structures led to a new family of crosslinked AIE supramolecular polymer gels, driven by hierarchical self-assembly involving coordination between the nitrogen and platinum atoms and host–guest interactions. These supramolecular polymer gels demonstrated gel–sol transitions responsive to various stimuli, including temperature, competing guest molecules, halides, and reversible “on-off” fluorescence. With their multifaceted response characteristics, it is anticipated that these crosslinked AIE supramolecular polymer gels will have a major impact on a number of sectors, including drug delivery, biological imaging, and molecular sensors.

The pillar[5]arene (P2) was able to coordinate with Al^3+^ ions, forming a supramolecular polymer metallogel that exhibited light blue AIE in the DMSO/H_2_O binary solution [60]. This gel showed responsiveness to multiple external stimuli, including temperature, guest compounds, ions (Fe^3+^ ions, F^−^ anions), acids (trifluoroacetic acid, TFA), and bases (triethylamine, TEA). The LODs ranged from 4.39 × 10^−9^ to 1.82 × 10^−7^ mol/L for Fe^3+^ ions, F^−^ anions, TFA, and TEA. The gel could detect Fe^3+^ ions, and Fe^3+^ ions coordinated metallogel could detect F^−^ anions through cation–π interactions and competitive coordination. The value of LOD for Fe^3+^ ions in the gel was 4.39 × 10^−9^ mol/L, while the LOD for F^−^ anions in the Fe^3+^ ion-coordinated metallogel was approximately 2.75 × 10^−8^ mol/L. Furthermore, the gel could detect TFA and TEA sequentially through protonation and deprotonation processes. Protonation enhanced the π–π stacking interaction between **P2** molecules. This material could be employed for multiple “write–erase–write” cycles, showcasing its versatility in detecting various stimuli.

Functional supramolecular polymer networks (SPNs) exhibit tunable luminescence properties [61]. These features set macrocyclic arene-based supramolecular gels apart from conventional pillar[*n*]arenes by expanding their range of functional building pieces. Developing soft materials based on pillar[*n*]arenes has captured the interest of many researchers. In 2019, Liu Z.J. and Yang Y.W. synthesized a monofunctionalized pillar[6]arene (P19) bearing a single terpyridine arm. They constructed a fluorescent SPN from P19, a TPE aggregation-induced emission generator G8, and Zn2+ ions as a coordinating linkage. The disruption of metal coordination and/or host–guest interactions by competitive binders such as TFA or pillar[5]arene, as well as heating, could weaken noncovalent interactions in the supramolecular polymer gels, inducing a gel–to–sol transition. These new architectural designs present encouraging paths for the creation of fluorescent SPN materials.

**Figure 9:**
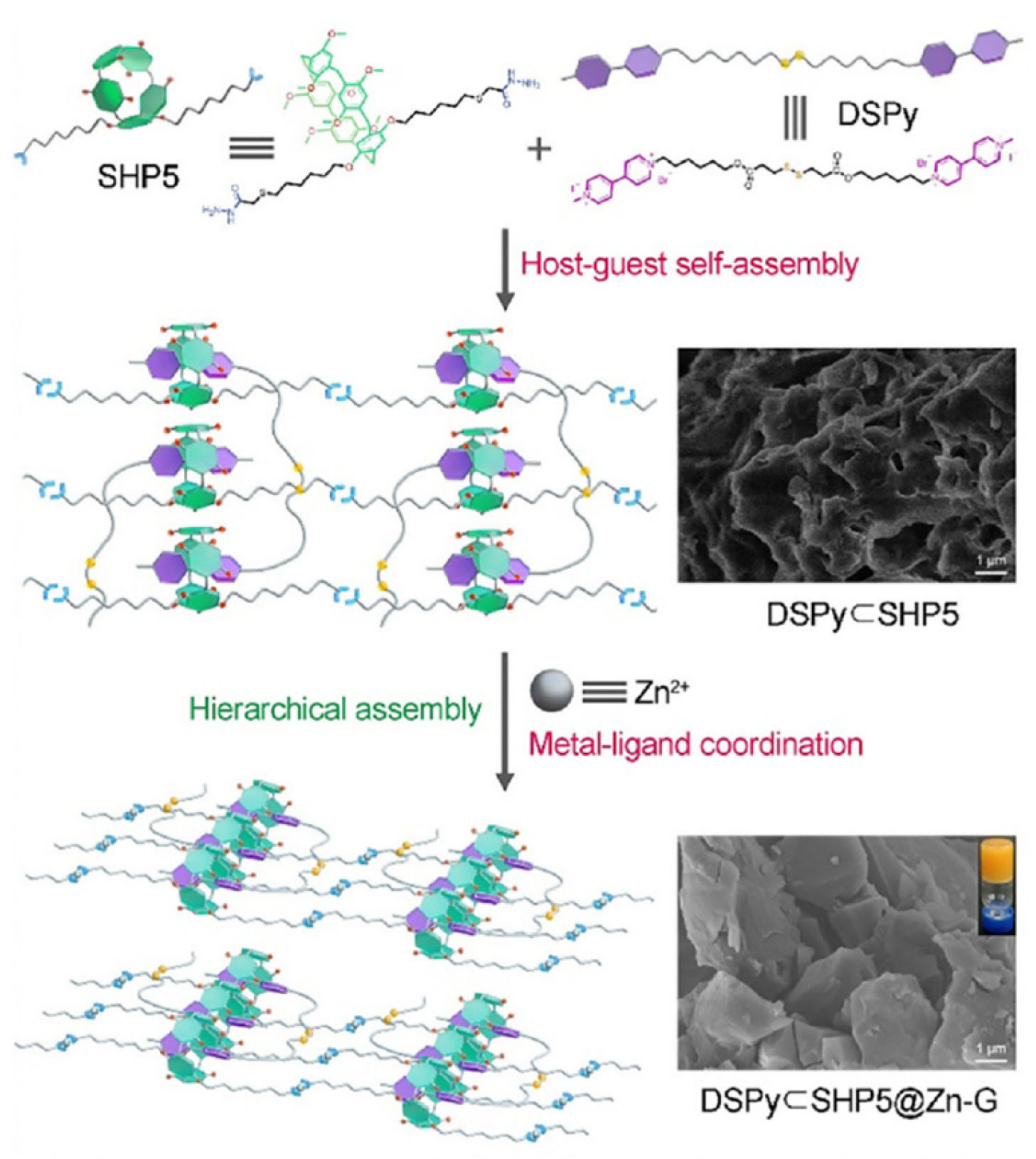
The development of the metallosupra-molecular gel DSPy⊂SHP5@Zn-G is schematically shown by the chemical structures of the host SHP5 and the guest DSPy, as well as by hierarchical host–guest recognition and metal ion coordination [62]. Copyright 2023, The Royal Society of Chemistry.

Zn^2+^ ions, a ditopic guest (**G9**), and a double-armed pillar[5]arene host (**P20**) assembled hierarchically to form another SPN [62]. Figure 9 shows that this assembly demonstrated sol–gel transition behaviors in response to a range of external stimuli, including as temperature, redox, pH variations, and competing guests. This assembly also functioned as a practical and easy-to-use test kit for the detection of OH^−^ anions. Given the advantageous properties of host–guest molecular recognition and the induction of metal–ligand coordination interactions, this work laid the groundwork for creating multi-stimuli-responsive metal supramolecular gels based on pillar[*n*]arene. The advancement of intelligent supramolecular materials with unique architectures and a variety of functions is greatly aided by these discoveries.

## 3. Conclusions and Outlooks

This review outlines the most recent studies in PSAs for developing fluorescence sensors capable of detecting environmental pollutants. In the last five years, notable advancements have been achieved in delineating the molecular structures, self-assembly mechanisms, photophysical properties, and sensing applications of these compounds. Recent PSAs-based sensors demonstrate enhanced capabilities in detecting various pollutants, offering superior solvent compatibility, porosity, versatility, and diverse detection capacities compared to traditional fluorescence sensors.

As highlighted above, fluorescence sensors have achieved notable success in designing and synthesizing innovative molecular structures and applying stimulus-responsive smart materials. However, the field faces new challenges and opportunities for future exploration and innovation. Firstly, the low concentration of analytes in real samples necessitates improvements in sensitivity and selectivity. Secondly, the fluorescence modulation mechanism under supramolecular regulation remains unclear, warranting further investigation into the luminescence mechanism, particularly concerning charge distribution and molecular architecture accumulation. Thirdly, the complexity of the constructed supramolecular systems often results in poor stability, posing challenges in practical applications. Simplifying these assemblies while maintaining or enhancing detection performance is critical for future research. Fourthly, developing supramolecular architectures that combine detection and adsorption functions requires further study, particularly in creating stable, reusable materials. Fifthly, there is a need for more research into PSAs capable of detecting multiple analytes simultaneously. Finally, while significant research has been conducted in developing supramolecular fluorescence sensors, their reliance on organic solvents hinders their applicability in biological and environmental systems.

In summary, fluorescence sensors based on PSAs containing metal coordination sites have been rapidly and continuously developed. We hope this review can help to promote design strategies for these sensors to better detect and adsorb environmental pollutants.

## Figures and Tables

**Table 1:** Summary of fluorescence sensors described in the section of single-stimulus responsiveness.

Pillar[*n*]arenes	Guests	Coordinated Metal Ions	Sensing Properties	Ref.
**P1**	**G1**	Ag^+^	Analyte: Ag^+^. Detection type: turn-on. *λ*_ex_/*λ*_em_: 310/470 nm. LOD: 1.50 × 10^−7^ mol/L. Liner range: 0–8.00 × 10^−6^ mol/L. Solvent: DMSO/H_2_O = 1/99.	[43]
**P2**	**−**	Ag^+^	Analyte: hydrazine hydrate. Detection type: turn-off. *λ*_ex_/*λ*_em_: 310/450 nm. LOD: 2.68 × 10^−8^ mol/L. Solvent: DMSO/H_2_O = 4/1.	[44]
**P3**	**−**	Hg^2+^	Analyte: Hg^2+^. Detection type: turn-off. *λ*_ex_/*λ*_em_: 332/486 nm. LOD: 4.30 × 10^−8^ mol/L. Solvent: DMSO/H_2_O = 1/1.	[45]
**P4**	**G2**	Hg^2+^	Analyte: Hg^2+^. Detection type: turn-on. *λ*_ex_/*λ*_em_: 312/388 nm. LOD: 3.00 × 10^−7^ mol/L. Liner range: 0–1.50 × 10^−5^ mol/L. Solvent: CHCl_3_/acetone/H_2_O = 1/4/495.	[46]
**P5**	**G3**	Hg^2+^	Analyte: Hg^2+^. Detection type: turn-off. *λ*_ex_/*λ*_em_: 510/565 nm. LOD: 7.17 × 10^−7^ mol/L. Solvent: H_2_O.	[47]
**P6**	**−**	Cu^2+^	Analyte: Cu^2+^. Detection type: ratiometric. *λ*_ex_/*λ*_em_: 333/384 nm. LOD: 1.85 × 10^−7^ mol/L. Solvent: CH_2_Cl_2_/CH_3_CN = 1/1.	[48]
**P7**	**−**	Cu^2+^	Analyte: Cu^2+^. Detection type: turn-off. *λ*_ex_/*λ*_em_ = 310/411 nm. Solvent: chloroform.	[49]
**P8**	**G4**	Zn^2+^	Analyte: nitrobenzene. Detection type: turn-off. *λ*_ex_/*λ*_em_: 295/460 nm. LOD: 1.66 × 10^−4^ mol/L. Liner range: 1.00–5.00 × 10^−5^ mol/L. Solvent: CH_3_CN/CHCl_3_ = 1/1.	[50]
**P9**	**−**	Fe^3+^	Analyte: Fe^3+^, L-Cys. Detection type: turn-off/on. *λ*_ex_/*λ*_em_: 375/535 nm. LOD: 6.06 × 10^−8^ mol/L (Fe^3+^); 1.00 × 10^−8^ mol/L (L-Cys). Solvent: cyclohexanol.	[51]
**P9**, **P10**	**P10**	Fe^3+^	Analyte: Fe^3+^, H_2_PO_4_^−^. Detection type: turn-off/on. *λ*_ex_/*λ*_em_: 375/530 nm. LOD: 7.54 × 10^−9^ mol/L. Solvent: cyclohexanol/H_2_O = 1/20.	[52]

**Table 2:** Summary of fluorescence sensors described in the section of dual-stimuli responsiveness.

Pillar[*n*]arenes	Guests	Coordinated Metal Ions	Sensing Properties	Ref.
**P11**	**G5**	Ag^+^, Hg^2+^	Analyte: Ag^+^, Hg^2+^. Detection type: ratiometric/turn-on. *λ*_ex_/*λ*_em_: 385/545 nm. LOD: 1.20 × 10^−8^ mol/L (Ag^+^); 5.00 × 10^−7^ mol/L (Hg^+^). Solvent: DMSO/H_2_O = 1/1.	[53]
**P12**, **P13**	**P13**	Fe^3+^, Cu^2+^	Analyte: Fe^3+^, Cu^2+^. Detection type: turn-off. *λ*_ex_/*λ*_em_: 330/460–560 nm. LOD: 8.53 × 10^−10^ mol/L (Fe^3+^); 4.57 × 10^−8^ mol/L (Cu^2+^).	[54]
**P14**	**−**	Cu^2+^	Analyte: nitroaromatics, Fe^3+^. Detection type: turn-off. *λ*_ex_/*λ*_em_: 380/487 nm. LOD: 3.90 × 10^−7^ mol/L (Fe^3+^); 4.90 × 10^−6^ mol/L (nitroaromatics). Liner range: 0–1.20 × 10^−3^ mol/L (Fe^3+^); 0–1.80 × 10^−4^ mol/L (nitroaromatics). Solvent: DMF/H_2_O = 1/4.	[55]
**P15**	**G6**	Cu^2+^, Hg^2+^	Analyte: Cu^2+^, Hg^2+^. Detection type: turn-off. *λ*_ex_/*λ*_em_: 293/324 nm. Solvent: CH_3_CN/water = 1/1.	[56]
**P2**, **P16**	**P16**	Fe^3+^	Analyte: Fe^3+^, F^−^. *λ*_ex_/*λ*_em_: 290/470 nm. LOD: 1.02 × 10^−10^ mol/L (Fe^3+^); 9.79 × 10^−9^ mol/L (F^−^). Liner range: 0–1.18 eq. (Fe^3+^); 0–0.86 eq. (F^−^). Solvent: cyclohexanol/H_2_O = 3/17.	[57]
**P17**	**G7**	Eu^3+^, Tb^3+^	Analyte: cyanide, perchlorate. Detection type: turn-on. *λ*_ex_/*λ*_em_: 290/470 nm. LOD: 5.96 × 10^−8^ mol/L (cyanide); 3.36 × 10^−6^ mol/L (perchlorate). Solvent: DMSO/H_2_O = 2/1.	[58]

**Table 3:** Summary of fluorescence sensors described in the section of multi-stimuli-responsiveness.

Pillar[*n*]arenes	Guests	Coordinated Metal Ions	Sensing Properties	Ref.
**P18**	**−**	Pt^2+^	Analyte: temperature, competitive guest molecules, halides. *λ*_ex_/*λ*_em_: 336/500 nm. Solvent: acetone/water = 1/19.	[59]
**P2**	**−**	Al^3+^	Analyte: Fe^3+^, F^−^, trifluoroacetic acid, triethylamine. Detection type: turn-off/on. *λ*_ex_/*λ*_em_: 380/470 nm. LOD: 4.39 × 10^−9^ mol/L (Fe^3+^); 2.75 × 10^−8^ mol/L (F^−^); 1.80 × 10^−5^ mol/L (trifluoroacetic acid); 1.80 × 10^−5^ mol/L (triethylamine). Solvent: DMSO/H_2_O = 4/1.	[60]
**P19**	**G8**	Zn^2+^	Analyte: competitive binding agents, trifluoroacetic acid, pillar[5]arene. *λ*_ex_/*λ*_em_: 349/385 nm. Solvent: CHCl_3_/CH_3_CN = 4:1.	[61]
**P20**	**G9**	Zn^2+^	Analyte: thermal, redox, pH, competitive guests. *λ*_ex_/*λ*_em_: 375/385 nm. Solvent: DMSO:H_2_O = 7/3.	[62]

## Data Availability

No new data were created or analyzed in this study. Data sharing is not applicable to this article.

## Abbreviations

AIE	Aggregation-induced emission
CGC	Critical gelation concentration
FRET	Fluorescence resonance energy transfer
DH	Hydrazine hydrate
RIR	Restriction of intramolecular rotation
LOD	Limit of detection
NAC	Nitro aromatic compound
PET	Photo-induced electron transfer
PNP	P-nitrophenol
SAIEE	Supramolecular assembly induced emission enhancement
SOF	Supramolecular organic framework
SPN	Supramolecules polymer network
TPE	Tetraphenylvinyl
TFA	Trifluoroacetic acid
TEA	Triethylamine
